# Preparation of Menthyl 3-amino-4-(2,4,5-trifluorophenyl) Butyrate and Investigation of its Hypoglycemic Activity

**DOI:** 10.2174/0115665240256416231120105956

**Published:** 2023-11-27

**Authors:** Xinmou Kuang, Minru Su, Hao Li, Xiaolan Sheng, Huan Cai, Shuilin Xie, Zhonghua Liu

**Affiliations:** 1 Zhejiang Collaborative Innovation Center for High-value Utilization of Byproducts from Ethylene Project, Ningbo Polytechnic, Ningbo Zhejiang 315800, China;; 2 School of Chemical Engineering, Ningbo Polytechnic, Ningbo Zhejiang 315800, China;; 3 SGS-CSTC Standards Technical Services (Ningbo) Co., Ltd. Ningbo Branch, Ningbo Zhejiang 315103, China;; 4 Department of Rehabilitation, Zhongshan People’s Hospital, Zhongshan Guangdong 528403, China;; 5 School of Biology and Biological, Engineering, South China University of Technology, Guangzhou Guangdong 510006, China

**Keywords:** 3-amino-4-(2,4,5-trifluorophenyl)butyrate, menthol, bioactivity, hypoglycemic activity, diabetes, sitagliptin analog

## Abstract

**Background:**

3-Amino-4-(2,4,5-trifluorophenyl) butyric acid has potential pharmacological effects in promoting insulin secretion. Menthol promotes drug transdermal absorption and hypoglycemic effects.

**Objective:**

The objective of the study was to combine the 3-amino-4-(2,4,5-trifluorophenyl) butyric acid and menthol to develop a new candidate drug molecule that can be used as a hypoglycemic drug in type II diabetes.

**Methods:**

In this study, the molecular structure of 3-amino-4-(2,4,5-trifluorophenyl) butyric acid in sitagliptin was modified by replacing pyrazine imidazole with menthol. The structure of the target compound was characterized by nuclear magnetic resonance (NMR). The anti-diabetic activity of BHF in N000180 BKS.Cg-Dock7m+/ +Leprdb/Nju mice with spontaneous diabetes was preliminarily studied.

**Results:**

A potential multi-target drug molecule, 3-amino-4-(2,4,5-trifluorophenyl) butyrate (BHF), was synthesized by combining 3-amino-4-(2,4,5-trifluorophenyl) butyric acid and menthol. BHF is suitable for hyperglycemic mice and has a significant hypoglycemic effect; the low dose of 10 mg/kg-1 started to be effective, and the high dose of 40 mg/kg-1 was more effective than the positive drug metformin.

**Conclusion:**

In this study, BHF has been synthesized and presented significant antidiabetic activities.

## 1 INTRODUCTION

Diabetes has the characteristics of high incidence, multiple complications, and serious adverse impact [1]. It involves two subtypes: type I diabetes and type II diabetes. According to the data released by the International Diabetes Federation in 2019, there were about 463 million adults with diabetes between the ages of 20 and 79 worldwide. It is predicted that there will be 578.4 million cases of diabetes in 2030 and 700.2 million cases in 2045 [2]. It is reported that patients with type II diabetes in the world account for 6.28% of the total population [3]. Type II diabetes is mainly caused by defects in insulin secretion and absorption, resulting in the inability of insulin function [4]. The incidence of type II diabetes, as the main clinical manifestation, tends to develop in the young population. Studies have found that the incidence probability of diabetic nephropathy, cancer, and other diseases in patients with type II diabetes is significantly increased [5]. In view of the harmfulness and preva-lence of diabetes, it is of great significance to establish innovative methods to develop new compounds and high-efficiency medicines to achieve the efficient prevention and treatment of type II diabetes.

The traditional treatment principle for type II diabetes is based on drug therapy aiming to mitigate symptoms, and hypoglycemic therapy is an effective approach to postponing diabetic nephropathy [2, 3, 6]. Patients with type II diabetes usually do not completely lose the capability for insulin production; in contrast, some patients even have high-level insulin production in the body. However, the functional effect of insulin is low; thus, the patients may have relatively deficient insulin in the body. The current treatment strategy focuses on stimulating the secretion of insulin in the body by taking some oral drugs. In the past decade, the primary medicines used for the treatment of type II diabetes included sulfonylureas, biguanides, thiazolidinediones, α-glucosidase inhibitors, dipeptidyl peptidase IV (DPP-4) inhibitors, thiazolidinediones (TZDs), sodium-dependent glucose transporter 2 (SGLT2) inhibitors, glucagon-like peptide-1 (GLP-1) receptor agonists, *etc*. [7-11]. The above medicines have made tremendous contributions to the treatment of type II diabetes, where they have been found to mainly achieve therapeutic effects by promoting insulin secretion or absorption in patients with type II diabetes, and indirectly control type II diabetes by protecting and enhancing the effect of endogenous incretin [12]. Other side effects mainly include hypoglycemia, edema, fractures, heart failure, ketoacidosis, and kidney damage [13-16].

Sitagliptin phosphate is a type II diabetes drug developed by Merck and approved by the FDA (Food and Drug Administration) in 2006. Sitagliptin’s chemical name is (3R)-3-amino-1-[3-(trifluoromethyl)-5,6,7,8-te-trahydro-1,2,4-triazolo[4,3-a]pyrazin-7-yl]-4-(2,4,5-tri-fluorophenyl)butan-1-one. 3-amino-4-(2,4,5-tri-fluoro-phenyl) butyric acid is one of the main structures in the molecular structure of sitagliptin. In this study, menthol has been used to replace the structure of pyrazine imidazole in sitagliptin. It effectively regulates blood sugar levels while reducing the risk of extreme hypo-glycemia. In recent years, combined administration of sitagliptin phosphate with metformin has been the most widely used hypoglycemic regimen in clinical practice [17].

As the predominant medical and health resource in China, traditional Chinese medicine (TCM) has unique advantages in the prevention and treatment of chronic diseases, while reducing the incidence of complications, and is especially suitable for the prevention and treatment of type II diabetes in the clinic practice. Menthol is one of the commonly used aromatic stimulants for resuscitation. Menthol is a cyclic monoterpenoid and the main active ingredient of menthol volatile oil [18]. In addition, menthol also has the penetration enhancer effect, which can also promote the absorption of insulin [19, 20]. Wei Qiumeng confirmed the hypoglycemic effect of menthol in mouse diabetes model experiments [21]. The menthol extracts can effectively reduce the fasting blood glucose levels in diabetic model mice, and it was concluded that menthol has a certain therapeutic effect on diabetes.

In this paper, the molecular structure of sitagliptin, 3-amino-4-(2, 4, 5-trifluorophenyl) butyric acid, has been modified, where the pyrazine imidazole structure has been replaced with menthol, and the combination drug has been used. Using a combinatorial molecular drug design strategy, *i.e*., combining Chinese and Western medicine, the molecular fragment 3-amino-4-(2, 4, 5-trifluorophenyl)butyric acid of sitagliptin phosphate was utilized to potentially promote the pharmacological effect of insulin secretion in conjunction with menthol in promoting drug transdermal absorption and hypoglycemic pharmacological effect [18, 21]. The 3-amino-4-(2, 4, 5-trifluorophenyl)butyric acid molecule was combined with menthol to synthesize BHF as a new compound. There are many similar cases where our research group has combined the Chinese and Western methods to develop a new candidate drug molecule that can be used as hypoglycemic drugs in type II diabetes to provide a reference for the molecular structure design [22-24].

In this paper, 2, 4, 5-trifluorophenylacetic acid was used as the raw material. Then, it was condensed with 2, 2-dimethyl-1, 3-dioxane-4, 6-dione to obtain a diketone compound. After the decarboxylation reaction, the obtained compound was combined with the addition reaction of *tert*-butyl carbamate to obtain enamine. After that, the asymmetric reduction has been conducted to obtain 3-amino-4-(2, 4, 5-trifluorophenyl) butanoic acid. Finally, 3-amino-4-(2, 4, 5-trifluoro-phenyl) butyric acid and menthol were combined to synthesize the target compound BHF. The antidiabetic activities of BHF have also been studied. Preliminary results have shown the compound BHF to present significant antidiabetic activities.

## 2 METHOD

### 2.1 Main Instruments

Nuclear magnetic resonance (NMR) instrument (AVANCE AV-400, Bruker Technology, Billerica, USA) (flow rate of 0.6 mL/min, detection at 215 and 254 nm) and high-performance liquid chromatography-mass spectrometer (Agilent 1200 series, Agilent Technologies, Santa Clara, USA) [source voltage (kV) positive mode: 4.00, negative mode: 3.5, sheath gas flow rate (arb): 45, aux gas flow rate (arb): 0, sweep gas flow rate (arb): 0; capillary voltage (V) positive mode: 35, negative mode: 35, capillary temp. (C): 250; tube lens voltage (V) positive mode: 110, negative mode: 200] were used.

#### 2.1.1 Preparation of Compound 3

The synthesis method reported previously has been employed [24]; in a 250 mL three-necked flask, 120 mL of tetrahydrofuran (Aladdin Bio-Chem, item no.: T103259) was first added, and then 10 g (52.6 mmol) of 2,4,5-trifluorophenylacetic acid (Aladdin Bio-Chem, item no.: B301174, Shanghai, China) was added. The mixture was stirred until it was completely dissolved. The temperature was set constant at 30℃, and 12.8 g (78.9 mmol) of N, N-carboxydiimidazole (Aladdin Bio-Chem, item no.: C109315, Shanghai, China) was slowly added four times, and then 0.96 g (7.89 mmol) of 4-dimethylaminopyridine (Aladdin Bio-Chem, item no.: D355485, Shanghai, China) was added. After stirring for 2.5 h, 9.1 g (63.1 mmol) of Mylbauer's acid (Aladdin Bio-Chem, item no.: M334635, Shanghai, China) was added, the flask was heated up to 50℃, and the reaction was carried for 7 h to finish the reaction. The resulting reaction product was evaporated to remove the solvent, and 50 mL of 10% hydrochloric acid (Guangzhou Chemical Reagent Factory, item no.: CB11-AR, Guangzhou, China) was added to the remaining species, extracted twice with 200 mL of dichloromethane (Aladdin Bio-Chem, item no. D116152, Shanghai, China), and the mixture was combined. The organic phase was rotary evaporated to obtain light-yellow oil, which was directly used in the next reaction.

#### 2.1.2 Preparation of Compound 4

The synthesis method reported previously has been employed [25]. In a 500 mL three-necked flask, the product obtained in the previous step was dissolved in methanol (Aladdin Bio-Chem, item no.: B163018, Shanghai, China) (200 mL), and 2,4,5-trifluorophenyl-acetic acid (Aladdin Bio-Chem, Item No.: B301174, Shanghai, China) (1 mL) was added slowly. The reaction was stopped after heating and refluxing for 5 h. After rotary evaporation to remove methanol, water (100 mL) was added, followed by extraction with ethyl acetate (Aladdin Bio-Chem, item no.: E116138, Shanghai, China) (200 mL) two times. The organic phases were combined, and saturated sodium chloride solution (Aladdin Bio-Chem, item no. C111542, Shanghai, China) (100 mL) was used to wash twice, which was followed by drying with anhydrous sodium sulfate (Aladdin Bio-Chem, item no.: S112288, Shanghai, China) and being concentrated under reduced pressure to obtain 12.1 g of light-yellow viscous oil in a yield of 60.8%. It was directly used in the next reaction.

#### 2.1.3 Preparation of Compound 5

The synthesis method reported previously has been employed [25]. In a 250 mL three-necked flask, 120 mL of methanol (Aladdin Bio-Chem, item no.: M116129, Shanghai, China) was added. The light-yellow viscous oil (12.1 g, about 38.3 mmol) obtained from the last reaction step was added. The mixture was stirred until completely dissolved, and then *tert*-butyl carbamate (Aladdin Bio-Chem, item no.: B107136, Shanghai, China) (4.5 g, 38.3 mmol) was added, which was followed by the addition of p-toluenesulfonic acid (Aladdin Bio-Chem, item no.: T104293, Shanghai, China) (0.8 g, 4.6 mmol) and anhydrous magnesium sulfate (Aladdin Bio-Chem, item no.: M116443, Shanghai, China). The mixture was heated to reflux for 12 h. The reaction solution was filtered, and the solvent was removed by rotary evaporation to obtain a white solid, which was then recrystallized from a mixed solution of methanol and water to obtain 6.9 g of a white powder in a yield of 77.3%.

#### 2.1.4 Preparation of Compound 6

The synthesis method reported previously has been employed [25]. In a 1000 ml reaction kettle, 150 ml of tetrahydrofuran (Aladdin Bio-Chem, item no.: T103259, Shanghai, China) was added. Compound ***5*** (6.5 g, 28 mmol) obtained in the previous step was added and dissolved completely. About 65 mg of the asymmetric reduction catalyst synthesized according to the method in the literature was added [26, 27]. The pressure was set to 10 atmospheres, and the reaction was carried out at room temperature for 18 h. The solvent was removed by rotary evaporation, and the crude product was recrystallized with a mixed solution of ethyl acetate (Aladdin Bio-Chem, item no.: E116131, Shanghai, China) and *n*-hexane (Aladdin Bio-Chem, item no.: H100107, Shanghai, China), and then filtered and vacuum-dried to obtain a white color solid 5.2 g in a yield of 80.7%.

#### 2.1.5 Preparation of Compound BHF

In a 250 mL three-necked flask, menthol (Aladdin Bio-Chem, item no.: M105138, Shanghai, China) (7.2 g, 12.8 mmol), compound **6** (4.9 g, 15.4 mmol), dicyclohexyl carbodiimide (Aladdin Bio-Chem, item no.: D106074, Shanghai, China) (5.3 g, 25.6 mmol), and 4-dimethylaminopyridine (Aladdin Bio-Chem, item no.: D109207, Shanghai, China) (0.3 g, 2.5 mmol) were added. Then, 100 mL of dichloromethane (Aladdin Bio-Chem, item no.: D116152, Shanghai, China) solution was added, which was followed by stirring at room temperature for 5 hours. The TLC plates (Aladdin Bio-Chem, item no.: T116948, Shanghai, China) were used to monitor the reaction. After the reaction stopped, the reaction solution was filtered, and the filtrate was sequentially washed with 1 mol/L HCl (Guangzhou Chemical Reagent Factory, item no.: CB11-AR, Guangzhou, China), saturated NaHCO_3_ solution (Aladdin Bio-Chem, item no.: S112337, Shanghai, China), saturated NaCl solution (Aladdin Bio-Chem, item no.: N378200, Shanghai, China), and anhydrous sodium sulfate saturated solution (Aladdin Bio-Chem, item no.: S112276, Shanghai, China), and finally, the solvent has been spin-dried. Then, 30 mL of ethyl acetate (Aladdin Bio-Chem, item no.: E116131, Shanghai, China) was added to dissolve the reactants, and 10 mL of a 2.3 mol/L solution of hydrogen chloride in ethyl acetate (Aladdin Bio-Chem, item no.: E105166, Shanghai, China) was added dropwise. The reaction mixture was stirred and filtered at room temperature, the solvent was spin-dried, and isopropyl ether (Aladdin Bio-Chem, product number: I110454, Shanghai, China) was added to obtain 4.2 g of a powdery white solid in a yield of 83.3%. The melting point was 244.2℃-245.6℃.

### 2.2 Antidiabetic Experiments

#### 2.2.1 Main Drugs and Reagents

Metformin has been obtained from Asia Pacific Pharmaceutical Co., Ltd., Zhejiang, China. The experimental mice used in this study were N000180 BKS.Cg-Dock7m +/+Leprdb/Nju mice being 6-8 weeks old and male; they have been provided by Nanjing University-Nanjing Institute of Biomedicine, China. Portable blood glucose tester and testing strips were provided by Roche Pharmaceutical Co., Ltd, Shanghai, China. Animal experiments have been approved by the IACUC of Guangzhou Huateng Biomedical Technology Co., Ltd. (HTSW00362).

#### 2.2.2 Grouping and Dosing Design of High Glucose Mouse Model

The experimental mice were randomly divided into 5 groups according to their body weight, with 6 mice in each group, namely the model control group, the positive control group, the low-dose group, the medium-dose group, and the high-dose group. Dosing was started after one week of adaptive feeding. The mode of administration was intragastric administration, where the model control group was given oral saline, and the positive control group was given metformin (200 mg kg^-1^); in addition, there were different dose groups, *i.e*., the low-dose group (10 mg kg^-1^), the medium-dose group (20 mg kg^-1^), and the high-dose group (40 mg kg^-1^). The administration dose of each group was 10 mg kg^-1^. The frequency of dosing was once a day for two consecutive weeks.

#### 2.2.3 Glucose Tolerance Test

N000180 BKS.Cg-Dock7m+/+Leprdb/Nju mice were divided into a hyperglycemia control group, metformin metformin-positive control group, and three sample dose groups with 6 animals in each group. After 5 hours of fasting, the sample dose groups were given high, medium, and low doses of the testing substances, respectively, and the hyperglycemic control group was given normal saline. About 20 minutes later, each mouse was given glucose 2.0 g kg^-1^ orally. Retro-orbital blood was collected to measure blood glucose values at three time points of 0, 0.5, 1, and 2 hours after glucose administration, and the area under the blood glucose curve at three time points was compared among dose groups and the hyperglycemia model control group.

### 2.3 Statistical Processing of Data

The data have been expressed as mean ± standard deviation, and the t-test was used for intergroup comparison. *p* < 0.05 was considered significantly different.

## 3 RESULTS

### 3.1 Synthesis and Structural Characterization of Compound BHF

**Fig. (1):**
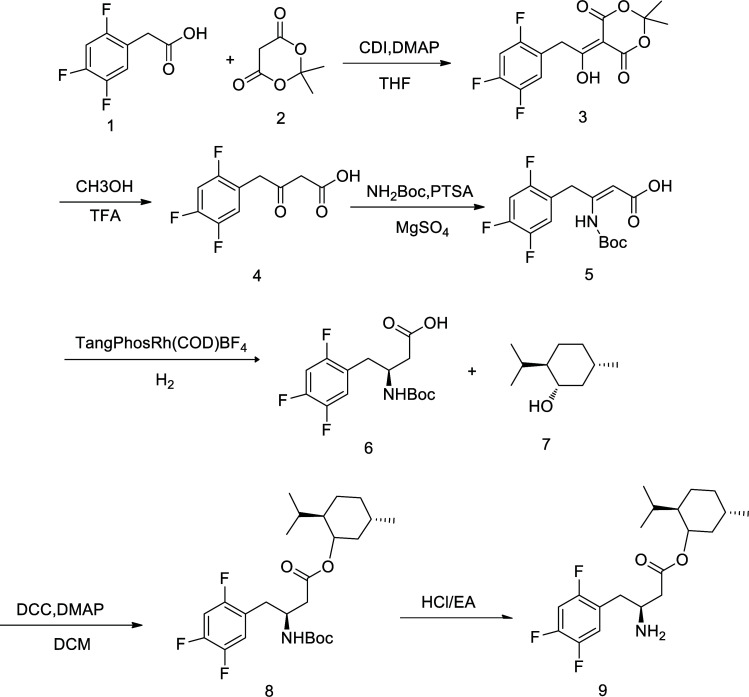
Scheme of synthesis of BHF.

The synthetic route is shown in Fig. **1**. Through hydrogen spectroscopy and carbon spectrometry, the compound was found to contain amino groups, ester groups, and other groups, and the molecular weight was 371.1, indicating the synthesized compound as the target compound. Figure S1 shows the ^1^H NMR (400 MHz, MD_3_OD) spectrum of BHF; ^1^H NMR (400 MHz, MD_3_OD) *δ* 7.38 – 7.27 (m, 1H, Ar-H), 7.22 (td, 1H, *J* 10.0, 6.6 Hz, Ar-H), 4.71 (td, 1H, *J* 10.9, 4.4 Hz,CHOH), 3.92 – 3.81 (m, 1H,CH-NH_2_), 3.16 – 2.97 (m, 2H,CH_2_), 2.79 – 2.62 (m, 2H,CH_2_), 1.94 – 1.81 (m, 2H,CH_2_), 1.75 – 1.66 (m, 2H,CH_2_), 1.48 – 1.33 (m, 2H,CH_2_), 1.16 – 1.04 (m, 1H,CH), 1.01 – 0.85 (m, 8H,(CH_3_)_2_CHCH), 0.78 (d, 3H, *J* 7.0 Hz,CH_3_). Figure S2 shows the ^13^C NMR (126 MHz, MD3OD) spectrum of menthyl BHF; ^13^C NMR (126 MHz, MD_3_OD) *δ* 16.67, 21.06, 22.34, 24.45, 27.47, 32.33, 32.63, 35.25, 36.54, 41.82, 48.22, 76.72, 107.05, 120.22, 120.59, 148.21, 151.01, 158.00, 171.15 ESI-MS m/z 372.1 [M+H^+^]; IR (KBr), ν/cm^-1^: 3403.24, 2954.84, 2927.84, 1739.73, 1286.47, 1232. 47.

### 3.2 Glucose Tolerance Test Results

The data in Fig. (**2**) shows that under the experimental conditions, the experimental system was essentially stable and reliable through the validation of the model control and positive control. The high-dose drug had a significant hypoglycemic effect on +/+Leprdb/Nju hyperglycemic mice. Moreover, the hypoglycemic effect of the high-dose drug was significantly better than that of the positive control drug metformin (200 mg kg^-1^ per day).

## 4 DISCUSSION

In recent years, there have been many studies on the design and synthesis of lead drug molecules for hypoglycemic drugs. On the basis of the molecular structure of glucokinase, Liang Jialong used the method of comparative molecular force field analysis to construct a three-dimensional structure-activity relationship model of glucokinase agonist, and constructed a compound molecule with good glucokinase agonist activity [28]. Zhang Chenglu reported the broad-spectrum pharmacological activities of 1, 2, 4-triazole and 1, 3, 4-thiadiazole, and combined them with active ferrocene, pyridine, furan, and other structures to design and synthesize novel molecules, including 1, 2, 4-triazole and 1, 3, 4-thiadiazole bis-heterocycle-modified sulfide amide derivatives, where the lead drugs with excellent activities have been screened [29]. These methods have relatively high uncertainties in obtaining lead candidates, and need to be screened on the basis of synthesis of a large number of lead molecules, and their efficiency is relatively low.

In this paper, using the combinatorial molecular chemistry design strategy, the menthol structure was introduced to the basic structure of sitagliptin, a key intermediate with well-documented efficacy, through the combination of Chinese and Western medicines. As a common TCM herb, menthol has relatively well-documented pharmacological effects, potential antidiabetic activities, and plays a role in promoting transdermal absorption. Therefore, 3-amino-4-(2, 4, 5-trifluorophenyl) butyric acid, the key intermediate of sitagliptin, was combined with menthol to synthesize BHF. The lead compound obtained by this method could have a high probability of obtaining good activities, which may improve the efficiency of lead drug screening.

The synthetic method of 3-amino-4-(2, 4, 5-trifluorophenyl)butyric acid, the key intermediate of sitagliptin, has been essentially carried out on the basis of the synthetic route recently reported by Merck and company (USA). Dooseop *et al.* reported the first-generation synthetic route of sitagliptin, which was relatively expensive and required relatively harsh experimental conditions, including −78℃ and −30℃ temperature conditions [30]. Hansen *et al.* first carried out asymmetric hydrogenation of β-ketoester with a chiral ruthenium phosphide catalyst to synthesize a chiral secondary alcohol, and then converted it into a chiral secondary amine [31]. This route involved many reaction steps, and the cost of industrial production was expected to be relatively high. The third-generation route reported by Merck and company firstly introduced an amino group in the reaction of (S)-2-amino-2-phenylacetamide, and then asymmetrically catalyzed hydrogenation by platinum oxide to obtain the R-type isomer, and finally, the protective group was removed under the action of a catalyst [32]. The expensive catalyst employed in this route leads to a relatively high synthesis cost. The method reported by Liu *et al.* requires the use of the Grignard addition reaction. Since the Grignard reaction is demanding and the yield is difficult to guarantee, it is expected that the scale-up reproduction would be challenging [33].

In this work, ethyl 2,4,5-trifluorobenzoate was used as the raw material and condensed with 2, 2-dimethyl-1, 3-dioxane-4,6-dione to obtain a diketone compound. After the decarboxylation reaction, the product was combined with the addition reaction of *tert*-butyl carbamate to obtain enamine, which was followed by asymmetric reduction to obtain 3-amino-4-(2, 4, 5-trifluorophenyl) butyric acid, and then the key intermediate of sitagliptin. In the synthetic process of the key intermediate of 3-amino-4-(2, 4, 5-trifluoro-phenyl) butyric acid in this work, the reaction process was relatively mild compared to other reported reaction conditions; the price of the reagents used was relatively suitable, and the reaction yield of each step was relatively stable, making this method suitable for subsequent scale-up and reproduction. Finally, 3-amino-4-(2, 4, 5-trifluorophenyl) butyric acid and menthol were combined to synthesize the target compound BHF, a lead drug candidate with potential hypoglycemic activities. The results have shown the target compound to be suitable for hyperglycemic mice having a significant hypoglycemic effect; the low dose of 10 mg/ kg-1 started to be effective, and the high dose of 40 mg/ kg-1 was more effective than the positive drug metformin.

The structure of the target compound was characterized by NMR, and the structural accuracy of the lead compound BHF synthesized in this work was confirmed.

## CONCLUSION

Based on the structure of sitagliptin’s molecular fragment 3-amino-4-(2, 4, 5-trifluorophenyl) butyric acid, this work has carried out structural modification; a potential multi-target prodrug molecule, 3-amino-4-(2, 4, 5-trifluorophenyl) butyrate (BHF), has been synthe-sized by combining 3-amino-4-(2, 4, 5-trifluorophenyl) butyric acid with menthol. The glucose tolerance test of hyperglycemic mice showed the target compound to have a significant hypoglycemic effect on N000180 BKS.Cg-Dock7m +/+Leprdb/Nju hyperglycemic mice. Under the high experimental dose (40 mg/ kg^-1^), the hypoglycemic effect of the obtained compound was better than that of the positive drug, metformin (200 mg kg^-1^ per day), which preliminarily validated the effective-ness of the theory of the combination molecular chemical design of prodrug molecule BHF. In the future, more in-depth research on the pharmacology, toxicology, and metabolism of this compound can be carried out. The lead drug candidate design strategy reported in this paper can also provide a reference for the theoretical design and implementation of potential related drug candidate molecules in other fields.

## Figures and Tables

**Fig. (2):**
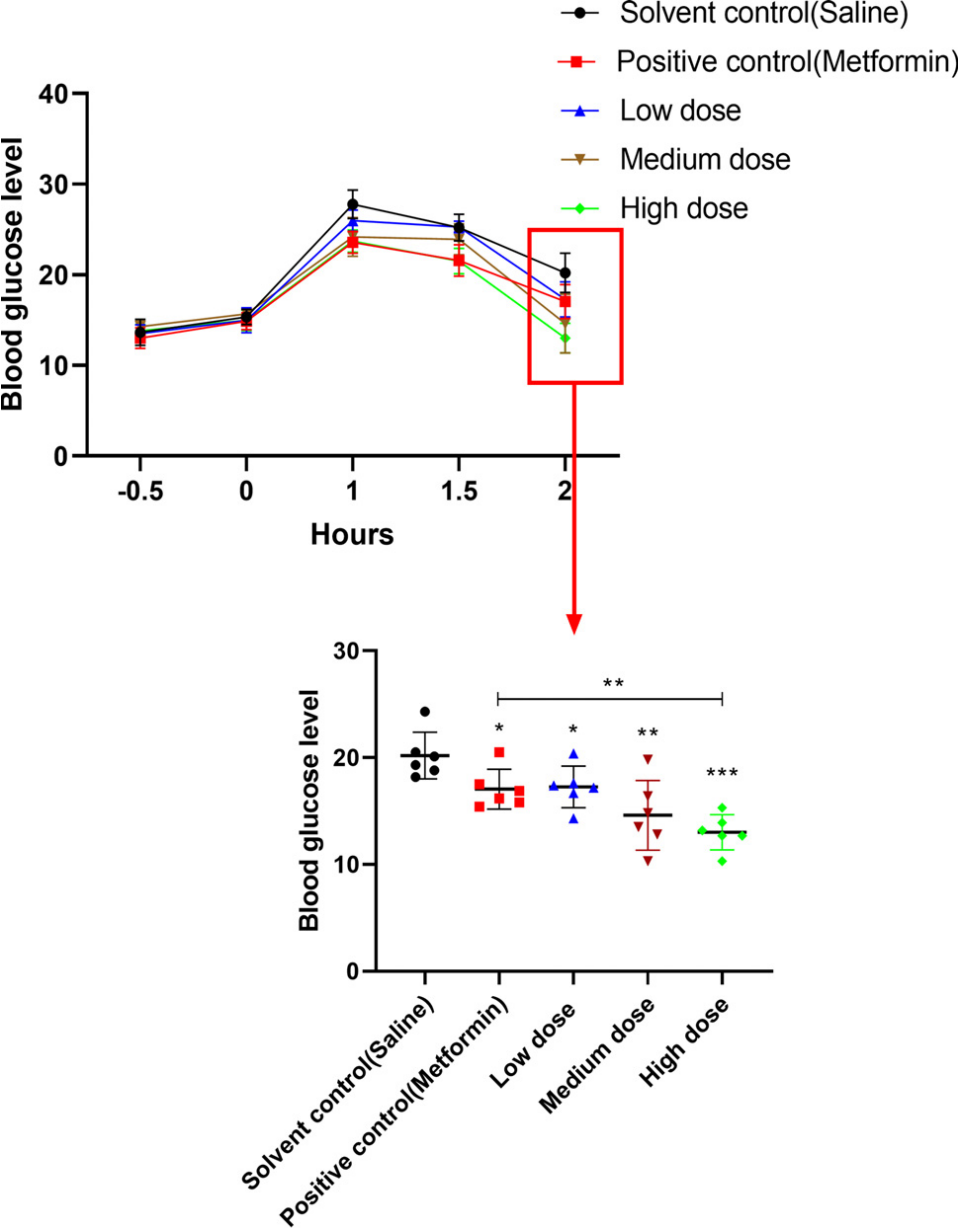
The area under the curve value of the glucose tolerance of hyperglycemic mice in each treatment group. *p < 0.05,**p < 0.01,***p < 0.001.

## Data Availability

The data and supportive information are available within the article.

## LIST OF ABBREVIATIONS

NMR: Nuclear magnetic resonance
DPP-4: Dipeptidyl peptidase IV
TZDs: Thiazolidinediones

## SUPPLEMENTARY MATERIAL

Supplementary data [^1^H NMR and ^13^C NMR spectra of menthyl 3-amino-4-(2, 4, 5-trifluorophenyl) butyrate] are available free of charge at http://jbcs.sbq.org.br as PDF file.
